# Effect of Heating Temperature on Ammonia Emission in the Mainstream Aerosols from Heated Tobacco Products

**DOI:** 10.3390/toxics10100592

**Published:** 2022-10-06

**Authors:** Takumi Yamamoto, Yoshika Sekine, Koki Sohara, Satoshi Nakai, Yukio Yanagisawa

**Affiliations:** 1 Graduate School of Science, Tokai University, Hiratsuka 259-1292, Japan; 2 School of Science, Tokai University, Hiratsuka 259-1292, Japan; 3 Graduate School of Earth and Environmental Sciences, Tokai University, Hiratsuka 259-1292, Japan; 4 Graduate School of Environment and Information Sciences, Yokohama National University, Yokohama 240-8501, Japan; 5 The University of Tokyo, Bunkyo 113-0033, Japan

**Keywords:** ammonia, aerosol, heated tobacco products, emission, gas-particle distribution

## Abstract

Heated tobacco products are devices that deliver nicotine into the body via inhalation of the mainstream aerosols generated during direct and/or indirect heating of tobacco leaf material. Ammonia in aerosols potentially increases the alkalinity and, therefore, the proportion of free nicotine for easy absorption. Meanwhile, ammonia can be a cause of adverse health effects when involved in the aerosols. This study aimed to grasp the emission behaviour of ammonia in the mainstream aerosols generated from four kinds of devices that employ different heating temperatures from 40 to 350 °C. The aerosols were generated by a vaping machine following the CRM 81 puffing protocol. Ammonia in the forms of gas and particles was trapped in 5 mM oxalic acid and subsequently determined by ion chromatography. The results showed that the total emission amount of ammonia increased with an increase in the heating temperature regardless of the device used. The gas-particle distribution of ammonia also depended on the heating temperature; gaseous ammonia was only found in the device with 40 °C of the heating temperature. These results show that ammonia in the mainstream aerosols was emitted from a common thermal process, probably thermal extraction in water vapour from a tobacco leaf.

## 1. Introduction

Heated tobacco products, also referred to as heat-not-burn tobacco products, are electrically operated devices that deliver nicotine into the human body via inhalation of the mainstream aerosol generated during the direct and/or indirect heating of tobacco leaf material [1,2,3,4]. These new products are being marketed as a less hazardous alternative to conventional combustible cigarettes for both smokers and non-smokers because (1) the tobacco leaf is heated without burning to generate nicotine-containing aerosols; (2) the nicotine-containing aerosols are generated during puffing only; and thus, (3) the emission of side-stream smoke is reduced or eliminated [1,2,3,4]. However, the use of these new products is still controversial regarding its harmful and addictive effects due to a lack of sufficient studies on the biological effects caused by the mainstream aerosol [5,6]. Therefore, the potential adverse health effects of these products should be carefully evaluated by investigating the chemical and physical properties of aerosols.

Nicotine is a natural and principal alkaloid in tobacco leaves. Since nicotine has two ionisable moieties, which are individually centred on the nitrogen atoms of pyridine and pyrrolidine heterocyclic rings, it can exist in a deprotonated form [Nic H_2_]^2+^, monoprotonated form [Nic H]^+^, and free base form [Nic] depending on pH [7,8,9].

[Nic H_2_]^2+^   ⇄   [Nic H]^+^   ⇄   [Nic]

p*K*_a1_ = 3.22     p*K*_a2_ = 8.11 (1)

The inhaled nicotine species is absorbed across biological membranes. In contrast to free nicotine, the protonated nicotine form poorly passes membranes, and it is therefore, believed to be less easily absorbed in the lungs [8,10]. When pH is greater than p*K*_a2_ =8.11, the free nicotine can be a dominant species. Ammonia has been known to exist in cigarette smoke and to potentially increase the alkalinity. Although denied by tobacco companies, numerous papers claimed ammonia-forming compounds, such as diammonium phosphate and urea, which have been occasionally added to certain brands of combustible cigarettes to boost nicotine delivery to smokers by increasing the alkalinity and, therefore, the amount of free nicotine [7,8,11,12].

Meanwhile, the mainstream aerosol generated by the heated tobacco products consists primarily of “water droplets,” which contain glycerine and/or propylene glycol, that functions as an aerosol former [13,14] and thus is fundamentally different in origin and chemical and physical composition when compared with conventional combustible cigarette smoke [13,14]. Ammonia is soluble in water, giving a basic liquid that contains solvated molecule (NH_3_), ammonium ion (NH_4_^+^), and hydroxide ion (OH^−^).

NH_3_ + H_2_O ⇄ NH_4_^+^ + OH^−^ (2)

Given that ammonia dissolves into the water droplets, the ammonium ion can couple with counter anions to form ammonium salts that distribute in the particle phase. The heated tobacco products do not emit side-stream smoke, but smokers exhale aerosols into the atmosphere. The inhalation of ammonia may cause nasopharyngeal and tracheal burns, bronchiolar and alveolar oedema, and airway destruction, resulting in respiratory distress or failure [15]. Therefore, the Occupational Safety and Health Administration (OSHA) has set a short-term (15 min) exposure limit of 35 ppm (24 mg m^−3^) for ammonia in the workplace. The National Institute of Occupational Safety and Health (NIOSH) has recommended that the ammonia level in workroom air must be limited to 50 ppm (35 mg m^−3^) for 5 min of exposure [16]. Ammonia’s odour threshold is sufficiently low to provide adequate warning of its presence at 5 ppm (3.5 mg m^−3^) [15,16].

According to Schaller et al. [17], ammonia was found in the mainstream aerosol generated from one heated tobacco product. However, the emission mechanism and status of ammonia in the aerosol were not fully investigated. At present, Japan is one of the largest consumer countries of these new products, with a variety of devices that employ different heating temperature from 40 to 350 °C. In this study, we aimed to grasp the emission behaviour of ammonia in the mainstream aerosols generated from these commercially available devices in Japan and found the effect of heating temperature on the emission amount and gas/particle distribution of ammonia.

## 2. Materials and Methods

### 2.1. Heated Tobacco Products

All devices used for the purposes of this study were purchased from a retail tobacco store in Japan. Table 1 summarizes the devices used; their heating temperature, flavour type, and operation mode; and the corresponding abbreviations used in this study. Device “A” consists of a rechargeable battery, a heating blade in the battery body, and a consumable tobacco stick. The tobacco leaves in the stick are impregnated with aerosol formers. The aerosol is generated by direct heating tobacco leaves with the blade at approximately 350 °C. Regular and menthol flavour sticks were used in this study. Device “B” consists of a rechargeable battery, cartridge, and tobacco capsule. The aerosol is generated by heating liquid in the cartridge containing aerosol formers and passing this through the tailor-made tobacco capsule at approximately 40 °C. This device is thus categorized as a “low-temperature-type” device, in contrast to others. Device “C” consists of a rechargeable battery, a heating furnace in the battery body, and a tobacco stick. The aerosol is generated by direct heating tobacco leaves in the furnace at approximately 200 °C. Only the device “C” has a “rapid heating” mode as an option to strengthen the taste of tobacco for smokers. Device “D” consists of a rechargeable battery and a tobacco stick that has a metal heating element placed at the core of the tobacco stick. The aerosol is generated by direct heating of the tobacco leaves by the inductively heated metal element at approximately 350 °C.

### 2.2. Experimental Setup

The mainstream aerosols were generated from all devices using an LM4E Linear Vaping Machine for E-cigarettes (Borgwaldt KC, GmbH, Germany) [18], following the Cooperation Centre for Scientific Research Relative to Tobacco (CORESTA) Recommended Method 81 (CRM81) [19]. As specified in the method, the puffing parameters were set at 55 mL puff volume, 3 s puff duration, and 30 s puff frequency, and a “Rectangle” puffing profile was used with or without installation of a Cambridge filter pad (44 mm diameter, Whatman, Little Chalfont, UK), which is used for the separation of the gas and particle phases.

For the simple measurement of the pH of aerosols, a piece of universal pH indicator paper (Advantec, Tokyo, Japan, pH range: 0–14) was exposed to the mainstream aerosols from the outlet of the vaping machine without a Cambridge filter pad. After exposure to a total of 10 puffs from all devices, the colour change in the paper was visually determined.

**Figure 1:**
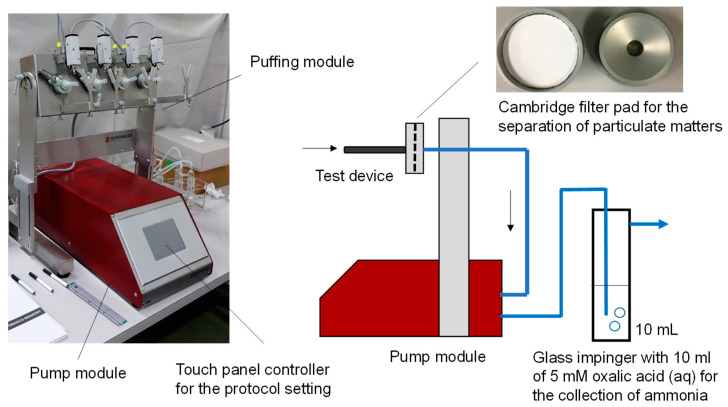
Schematic view of the vaping machine and sampling apparatus used in this study.

For the measurement of ammonia species in aerosol, a glass impinger filled with 10 mL of 5 mM oxalic acid solution was connected to the outlet of the vaping machine to trap ammonia emissions with a silicon tube with/without a Cambridge filter pad (Figure 1). A total of 10 puffs (55 mL × 10 puffs = 550 mL) of the mainstream aerosol were generated from devices A, C, and D, and then passed through the 5 mM oxalic acid solution to trap ammonia. Due to a lower emission of ammonia from device B, the amount was set at 50 puffs for this device only. The use of a backup impinger revealed no significant breakthrough during sampling. Ammonia trapped as ammonium ion was subsequently determined by ion chromatography after being filled up to 10 mL with 5 mM oxalic acid. The ion chromatography system consists of a Shimadzu LC-20AD pump, a conductivity detector, a Shimadzu COD-10A vp, a Shimadzu column oven, a CTO-10A vp, and a recorder. The following conditions were used: column, 4.6 mmφ × 150 mm, IC-C4 (Shimadzu, Kyoto, Japan); eluent, 5 mM oxalic acid at 1.0 mL min^−1^ (isocratic); automatic injection volume, 20 µL; and oven temperature, 313 K. A dilution series of ammonium ion in Milli-Q water at 0.0, 0.20, 0.50, 2.0, and 5.0 mg L^−1^ was prepared from ammonium sulphate and used for calibration (*r* = 0.996 for ammonia concentration versus peak area). All reagents were purchased from Kanto Chemicals (Tokyo, Japan). Three repeated measurements were conducted for all runs. After subtracting the mean blank reading of the storage trapping from the sample readings, the collection amount of ammonia (µg) was converted to the emission amount of ammonia in a puff volume, *E* (µg L^−1^):

*E* = *W*/*V* (3)

where *W* is the collection amount of ammonia (µg) and *V* is a total puff volume (0.55 L at 10 puffs and 2.75 L at 50 puffs). Since significant contamination was found in the storage blank, the limit of determination (LOD) was defined as a blank reading + 10σ_b_ of five storage blanks. The LOD resulted in 0.45 µg L^−1^ for 10 puffs and 0.18 µg L^−1^ for 50 puffs.

## 3. Results and Discussion

### 3.1. Aerosol pH and Total Ammonia Emission

**Figure 2:**
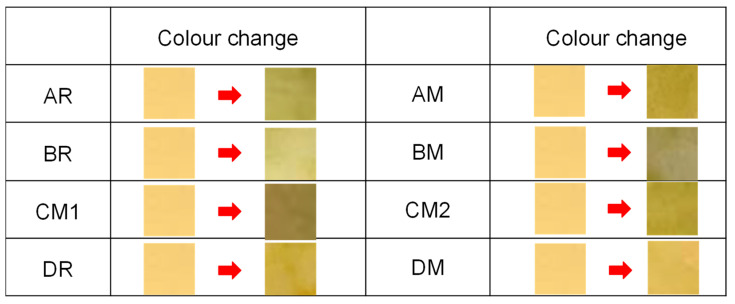
Colour changes in the universal pH indicator paper before and after exposure to 10 puffs of the mainstream aerosols generated from each heated tobacco product.

Because of the acid–base equilibrium of nicotine, the basic environment of aerosol is favourable for nicotine absorption into body. Figure 2 shows colour changes in the universal pH indicator before and after exposure to the mainstream aerosols. The colour turned from light green (pH 7) to dark green (pH 8–9) for every run, showing that the “water droplets” generated from the heated tobacco products were weakly basic, so that it is a favourable environment for the absorption of nicotine.

**Figure 3:**
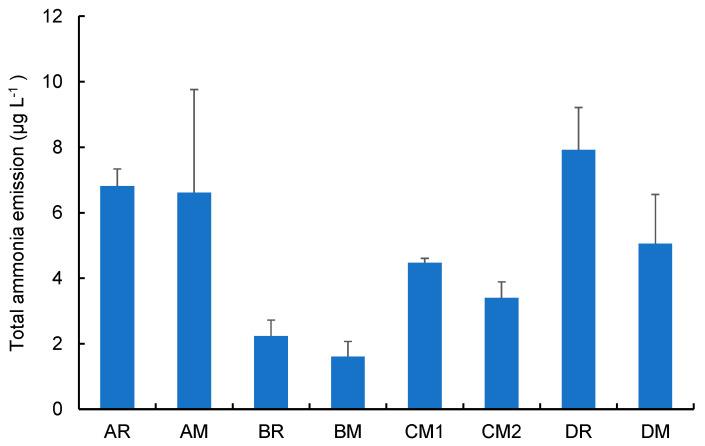
Analytical results on the total emission amount of ammonia (gas + particle forms) from each heated tobacco product (error bars shows standard deviations of triplicate measurements).

Figure 3 shows the arithmetic means of the total amount of ammonia emissions collected from all devices (the values were measured without the Cambridge filter pad). Error bar shows the standard deviations of three repeated runs. Greater emissions were found in the “high-temperature type” devices A and D: AR, 6.8 ± 0.52 µg L^−1^; AM, 6.6 ± 3.1 µg L^−1^; DR, 7.9 ± 1.3 µg L^−1^; and DM, 5.1 ± 1.5 µg L^−1^. The differences in the values between the regular and menthol flavour types were not significant (*t*-test). Schaller et al. [13] have determined the ammonia emissions from device A when employing the Health Canada Intense (HCI) protocol [20] and reported 15.6 µg/stick, which corresponds to 24 µg L^−1^ (55 mL of puff volume, 12 puffs). This difference from the previous report must be caused by the difference in the puffing protocol because the chemical composition of mainstream smoke varies with smoking conditions [21,22,23]. The HCI protocol was originally developed for traditional cigarettes with the puffing parameters 55 mL puff volume, 2 s puff duration, and 30 s puff frequency with a “Bell” puffing profile. Therefore, differences in the puff duration and puffing profiles might cause a deviation in the ammonia emission. On the contrarily, lower emissions were found in the “low-temperature-type” device B: BR, 2.2 ± 0.48 µg L^−1^ and BM, 1.6 ± 0.46 µg L^−1^, with no significant difference in flavour type. No difference was also found in the operation modes of device C: 4.6 ± 0.14 µg L^−1^ and 3.4 ± 0.48 µg L^−1^. Compared with short-term exposure limits set by OSHA (24 mg m^−3^ = 24 µg L^−1^) and NIOSH (35 mg m^−3^ = 35 µg L^−1^), these total amount of ammonia emissions were less than 1/3 of the limit values, suggesting no severe toxic effects on potential users of these devices.

**Figure 4:**
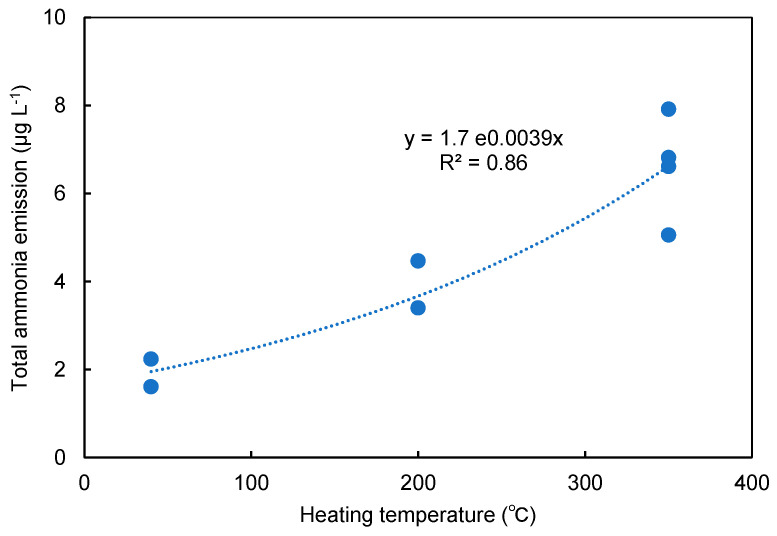
The relationship between the total emission amount of ammonia and the heating temperature of devices used in this study. Single exponential regression analysis; y = 1.7e^0.0039x^, *r*^2^ = 0.86.

Since the total ammonia emissions were in the order of devices A = D > C > B, they were plotted against the heating temperature of each device, as shown in Figure 4. Even though the heating temperature was cited from a commercial catalogue and/or technical information provided by each different manufacture, the total ammonia emission increased with an increase in the temperature. Thermal process can often be characterized by reaction temperature, and pre-exponential and activation energy in the Arrhenius equation with an exponential growth [24]. Therefore, a single exponential regression analysis was applied to the plots, and y = 1.7e^0.0039x^ was obtained with a significant coefficient of determination of 0.86. This suggests that ammonia in aerosols is emitted from a common thermal process regardless of the devices used in this study. Since there is no evidence on the addition of ammonia-forming compounds to every heated tobacco product, it is reasonable to think of the common thermal process as a thermal extraction of ammonia with water vapour from tobacco leaves during the aerosol-forming step in the devices. 

### 3.2. Gas–Particle Distribution of Ammonia

**Figure 5:**
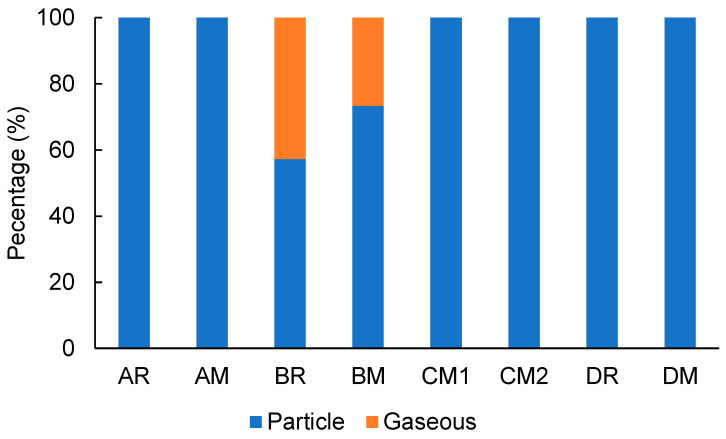
Percentages of particles of ammonia emitted from each heated tobacco product (average of three repeated measurements).

The ammonia emissions from the devices were also measured with a Cambridge filter pad that traps particulate ammonia species emitted from the devices. Figure 5 shows the percentages in the particle form of ammonia emitted from each heated tobacco product. No gaseous ammonia was found for devices A, C, and D (below LOD), so the ammonia species were presented in particle form in the aerosols generated from the “high-temperature-type” devices. Meanwhile, gaseous ammonia emission was only found for the “low-temperature-type” device B: BR, 0.95 ± 0.28 µg L^−1^ and BM, 0.42 ± 0.10 µg L^−1^. Additionally, the percentage of particles of ammonia resulted in 57% for BR and 73% for BM.

This difference is probably because of difference in the aerosol-forming processes. According to Tonokura and Hayashi [25], glycerine vapours generated during heating were responsible for the extraction of chemical components in tobacco leaves for direct heating-type devices such as devices A, C, and D. Therefore, ammonia species may immediately be extracted in glycerine vapour with potential counter ions to form salts, and the hygroscopic vapour subsequently grows with the amount of moisture to become mainstream aerosols. On the contrary, since only device B employs indirect heating of tobacco leaves with water vapours, ammonia species may be extracted with water and certain portions are emitted in gaseous form before forming salts. 

## 4. Conclusions

In this study, we aimed to grasp the emission behaviour of ammonia in the mainstream aerosols generated from heated tobacco products commercially available in Japan. Since the devices employ different heating temperatures from 40 to 350 °C, the effect of heating temperature on the amount of ammonia emissions and the gas/particle distribution were investigated. The results showed that the total emission amount of ammonia increased with an increase in the heating temperature regardless of the device used. The gas–particle distribution of ammonia also depended on the heating temperature; most of ammonia existed in the form of particles. These results show that ammonia in the mainstream aerosols were emitted from a common thermal process, probably thermal extraction in water vapour from tobacco leaves.

## Figures and Tables

**Table 1:** Heated tobacco products used in this study.

Device	Heating Temperature (°C)	Flavour Type	Operation Mode	Abbreviation
A	350	Regular	default	AR
		Menthol		AM
B	40	Regular	default	BR
		Menthol		BM
C	200	Menthol fresh	rapid heating	CM1
			default	CM2
D	350	Regular	default	DR
		Menthol		DM

## Data Availability

The data presented in this study are available within the article (tables and figures). The data presented in this study are available from the corresponding author upon request.
